# Optimizing dietary lipid use to improve essential fatty acid status and reproductive performance of the modern lactating sow: a review

**DOI:** 10.1186/s40104-016-0092-x

**Published:** 2016-06-07

**Authors:** David S. Rosero, R. Dean Boyd, Jack Odle, Eric van Heugten

**Affiliations:** The Hanor Company, Franklin, KY 42134 USA; Department of Animal Science, North Carolina State University, Raleigh, NC 27695 USA

**Keywords:** Essential fatty acids, Lactating sow, Subsequent reproduction, Supplemental lipids

## Abstract

Dietary lipid supplementation benefits the prolific and high-producing modern lactating sow. A comprehensive review of recent studies showed that lipid supplementation increases average daily energy intake, which is partitioned for lactation as indicated by greater milk fat output and improved litter growth rate. Recent compelling findings showed that addition of particular lipids during lactation improved the subsequent reproductive outcome of sows. Such benefits were related to the level of dietary essential fatty acids (EFA, linoleic acid, C18:2n-6; and α-linolenic acid, C18:3n-3) during lactation. Lactation diets without supplemental EFA resulted in a pronounced negative balance (intake minus milk output) of linoleic (−25.49 g/d) and α-linolenic acid (−2.75 g/d); which compromised sow fertility (farrowing rate < 75 % and culling rates > 25 % of weaned sows). This phenomenon seems to be increasingly important with advancing sow age because of a progressive reduction of body EFA pool over successive lactations. The net effect of supplemental EFA during lactation was to create a positive EFA balance, which improved the subsequent reproduction of sows. Adequate linoleic acid intake improved the proportion of sows that farrowed in the subsequent cycle (Farrowing rate (%) = [(−1.5 × 10^−3^ × linoleic acid intake (g/d)^2^) + (0.53 × linoleic acid intake (g/d)) + (45.2)]; quadratic *P* = 0.002, R^2^ = 0.997, RMSE = 0.031). In addition, increasing linoleic acid intake increased the number of pigs born in the subsequent cycle (total pigs born (n) = [(9.4 × 10^−5^ × linoleic acid intake (g/d)^2^) + (0.04 × linoleic acid intake (g/d)) + (10.94)]; quadratic *P* = 0.002, R^2^ = 0.997, RMSE = 0.031). Supplemental α-linolenic acid resulted in a rapid return to estrus (sows bred: sows weaned = 94.2 %; wean-to-estrus interval = 4.0 d) and achieved a high retention of pregnancy (sows pregnant: sows bred = 98 %). Collectively, we conclude that a minimum dietary intake of 10 g/d of α-linolenic acid, simultaneous with a minimum of 125 g/d of linoleic acid should be provided to ≥ 95 % of the sows; thereby, achieving a maximum sow reproductive efficiency through multiple mechanisms that include rapid return to estrus, high maintenance of pregnancy and large subsequent litter size in mature sows, that appear to be susceptible to EFA deficiency.

## The modern lactating sow

Improvements in swine genetics and management have resulted in a prolific (15.1 total pigs born per litter) and high-producing (11.5 pigs weaned per litter) modern sow (farms in the top 10 %) [1]. For larger and fast-growing litters, the demand for milk and nutrient output has increased substantially (Table 1). In 1985, it was estimated that the average sow produced 8.2 kg of milk/d for the nursing litter while in 2012 it is estimated that milk production can be as high as 9.2 kg/d for the elite sow nursing litters that grow at 2.35 kg/d. This is 34 % greater than the milk output estimated for the reference sow of the past which only produced 6.9 kg/d [2, 3].

**Table 1:** Comparison between the productive parameters of the elite modern lactating sow and the reference sow of the past

	1985^a^	2012^b^	
Item	Average sow	Average sow	Elite sow^c^
Total pigs born, n	11.2	13.4	15.1
Pigs weaned,^d^ n	8.6	10.3	11.5
Litter gain,^e^ kg/d	1.60	2.09	2.35
Milk production,^f^ kg/d	6.9	8.2	9.2
Nutrient output,^f^ g/d			
Lactose	385	458	512
Protein	379	450	501
Fat	526	626	699

^a^Source: [2, 3]

^b^Source: [1]

^c^Top 10 % of farms

^d^Lactation length: average sow 1985 = 25.1, average sow 2013 = 20.5, and elite sow 2013 = 22.3 d

^e^For the average sow of 1985, litter gain estimation used information provided by Noblet and Etienne [3]

^f^Milk production and nutrient output was calculated based on litter weight gain and litter size at weaning using a set of equations derived from Hansen et al. [40]

Development of the modern sow has also resulted in an animal with less body fat reserves and lower appetite [4]. Thus, sow feeding programs need to ensure optimal consumption of nutrients and energy to support this high level of milk production, but prevent large sow body weight (**BW**) loss and to maximize the long-term productivity of the sow. Optimal nutrient intake by the lactating sow becomes more challenging under certain management and environmental conditions. Exposure of sows to high ambient temperature results in physiologic and metabolic changes that impairs intestinal barrier function, increases oxidative stress and dramatically reduces nutrient intake; which leads to mobilization of body reserves to meet the nutrient deficiency [5–8].

Excessive mobilization of body tissues during lactation compromises the subsequent reproductive performance of sows [9]. King [10] found a correlation (R^2^ = 0.63) between the loss of lean body mass and wean-to-estrus interval (**WEI**). In addition, Hughes [11] concluded that body fat status was also a factor for optimum reproduction outcome and determined that back fat loss greater than 2 mm during lactation compromised the subsequent pig-output of sows. Thus, meeting the amino acids and energy needs during lactation is important for milk production, maintenance of body reserves, and reproduction outcome. Supplementation of lipids to lactation diets has been a nutritional strategy to benefit the lactating sow, especially those under heat stress conditions [12]. It is plausible that supplemental lipids have greater impact for the prolific and high-producing lactating sow because of the greater demand for milk production. This review summarizes the contemporary literature on the nutritional value of lipids for the modern sow, with special emphasis on compelling new findings regarding the impact of essential fatty acid adequacy.

### Lipid nutrition during lactation

Dietary lipids are extensively used in swine diets as sources of energy and essential fatty acids. While the energetic role of the former is known, the exact nature of the latter has only recently been evaluated. Potential benefits of dietary lipids for the lactating sow and progeny have been extensively studied over the last 30 years, but results from studies are inconsistent and benefits for the lactating sow need clarity. Earlier reviews [12, 13] suggest that potential benefits of supplemental lipids were evident only when sows experienced management or environmental challenges. An important statement of context was provided by Dr. B. G. Harmon (personal communication, 2015). Their experience at Purina Mills during the 1980–1990 timeframe was that improvements in lactating sow performance, arising from added lipid, were easier to demonstrate under field conditions, because many sow farm managers were limit feeding sows. This review considered published studies investigating the effects of dietary lipids on lactating sow performance, when the modern sow was used.

#### Nutritional value of lipid sources

Lipids, commonly referred to as fats or oils, are a group of substances found in plants and animal tissues that are insoluble in water, but soluble in non-polar solvents. Nutritionally, lipids are considered as a highly digestible energy source for pigs; however, this may differ between sources of lipid because of varying chemical composition, quality, and peroxidation status [14, 15]. Commercially available sources of lipids are often blended products, mainly restaurant by-products and rendered fats. Processed lipids (e.g. by-product lipids) can be exposed to peroxidation, which negatively affects nutrient digestibility, absorption capacity of the intestine, and gastro-intestinal health status [16, 17]. Considering the different factors that impact digestion and absorption rate of lipids, it is important to accurately determine the energy value of sources of lipid for diet formulation.

Powles et al. [18] described a prediction equation that is used by many nutritionists to estimate the digestible energy (DE) content of lipids with varying free fatty acid (FFA) levels and unsaturated to saturated (U:S) fatty acid ratios when fed to growing pigs. Using similar methods, Rosero and co-workers [19] recently determined apparent lipid digestibility in lactating sows for sources with varying FFA levels and U:S ratios and developed a prediction equation that more accurately estimate the DE content of lipids for the sow:

$$ \mathrm{D}\mathrm{E}\ \left(\mathrm{kcal}/\mathrm{kg}\right) = 8,381\ \hbox{--}\ \left(81 \times \mathrm{F}\mathrm{F}\mathrm{A}\right) + \left(0.4 \times \mathrm{F}\mathrm{F}{\mathrm{A}}^2\right) + \left(249 \times \mathrm{U}:\mathrm{S}\right)\ \hbox{--}\ \left(28 \times \mathrm{U}:{\mathrm{S}}^2\right) + \left(12.8 \times \mathrm{F}\mathrm{F}\mathrm{A} \times \mathrm{U}:\mathrm{S}\right);\ {\mathrm{R}}^2 = 0.741. $$

where FFA is the concentration of free fatty acids in the lipid (%) and U:S is the unsaturated to saturated fatty acid ratio.

Application of this prediction equation resulted in relatively small errors of prediction (residual divided by the predicted value; errors ranged from −4.7 to 2.0 %). In a similar manner as that described by Powles et al. [18], Rosero et al. [19] accurately estimated the DE content of lipids using chemical composition parameters; however, further improvement of this equation is warranted by using other factors (e.g. peroxidation status, correction for endogenous loss) that affect digestion and absorption of lipids.

#### Effect of supplemental lipids on sow and litter performance

Dietary lipid increases energy density of sow diets and has the advantage of having a low heat increment associated with digestion and metabolism [20]. Supplemental lipids are also believed to increase caloric intake of sows in spite of reduced feed intake stemming from external factors such as high temperatures [21, 22]. Because lactation is a physiological priority, greater caloric intake is partitioned into milk fat secretion, which may improve survival and growth of nursing piglets [12, 13]. The potential benefits of supplemental lipids for sow and litter performance were summarized using 12 references published from 1989 to 2012 [21–32]. The present review focused on average daily energy intake (**ADEI**), sow BW change, and litter gain as observations of interest (Table 2).

**Table 2:**
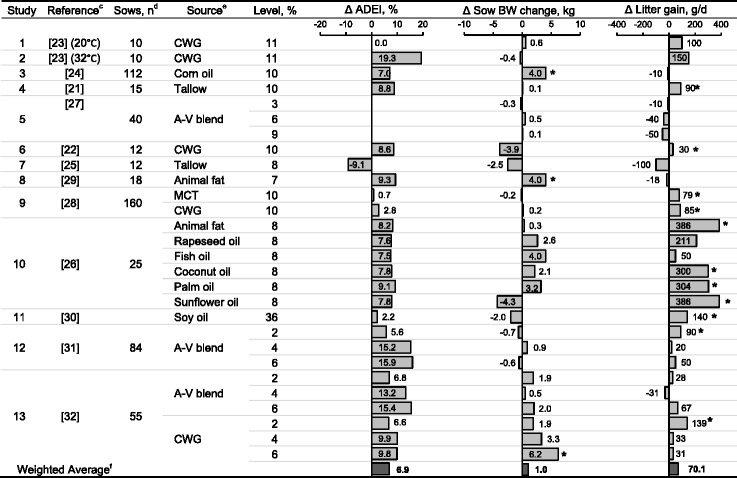
Overview of the studies that investigated the effects of lipid supplementation on sow lactating performance^a,b^

^a^Summary of the results from 13 published studies from 1989 to 2012 that were not included in previous reviews [12, 13] and reported on supplemental dietary lipids in lactating sows diets

^b^Estimated means represented by bars show the improvement of lipid supplementation relative to lactation diets containing no added lipids. Variables reported include: average daily energy intake (ADEI), sow body weight (BW) change, and litter daily gain

^c^References are presented in chronological order

^d^n refers to the number of observations per treatment and was used as the weighing factor to calculate the average of the response

^e^Lipid sources: choice white grease (CWG), animal-vegetable blend (A-V blend), and medium chain triglycerides (MCT)

^f^Weighted averages were calculated to account for the different sample sizes in each of the studies

*P < 0.05

In this analysis, ADEI averaged 15.9 Mcal metabolizable energy (ME) and ranged from 10.4 to 24.3 Mcal ME/d. Supplemental lipids improved ADEI in all but 3 of the 12 studies. The improvement in caloric intake was estimated to be 6.9 % (weighted average considering differences in sample size among studies) or 1.10 Mcal ME/d, which is in close agreement with the 1.24 Mcal ME/d improvement reported in an earlier review by Pettigrew and Moser [13]. This positive response on ADEI varied depending on the level of supplemental lipid, lipid source, and environmental conditions. Studies examined in the present review used supplemental lipid levels that ranged from 2 to 11 %, with only 2 studies investigating the impact of supplemental lipids on caloric intake in a dose-dependent manner [31, 32]. The change in ADEI, when lipid was supplemented to lactation diets, was described by Δ ADEI (%) = [− 0.46 + (supplemental lipid (%) × 4.5) + (supplemental lipid (%)^2^ × (− 0.34)); quadratic *P* < 0.001; R^2^ = 0.871; RSME = 18.2]. Although a total of 13 different sources of lipid were used in the studies reviewed, only 3 studies compared the effects of lipid source [26, 28, 32]. None of these studies reported significant differences on ADEI between sources. Moreover, it is expected that a greater benefit would be observed when sows experience heat stress because of the lower heat increment associated with digestion and metabolism of lipids [20]. The study conducted by Schoenherr et al. [23] supports this hypothesis.

The greater caloric intake by lipid fed sows slightly reduced sow BW loss during lactation by a weighted average of 1.0 kg. However, responses were inconsistent (19 positive and 9 negative responses) and only 3 studies reported significant improvements [24, 29, 32]. This positive response on sow BW loss depended on the genetic line (Landrace, but not Duroc sows responded positively) [24], and lipid source (added choice white grease, but not animal-vegetable blend) [32].

As reviewed in 1991, Pettigrew and Moser [13] suggested that supplemental lipids improved litter weight at weaning by 1.65 kg (80 g/d assuming a 21 d of lactation) when compared with diets without added lipids. In this review, supplemental lipids consistently (10 positive responses were significant) improved litter growth by a weighted average of 70.1 g/d. The elevated responses reported by Lauridsen et al. [26], contributed substantially to this weighted average for daily litter gain. The positive benefit of supplemental lipid on litter weight gain was more evident in later studies (year 2000 and beyond). Potential benefits of supplemental lipids on piglet survival were also explored in the reviewed studies, but the response was inconsistent (data not shown).

#### Effect of supplemental lipids on sow milk

Supplemental lipids may increase milk fat output while reducing the energetic cost for the relatively high de novo fatty acid synthesis that is noted in the sow [33]. A thorough description of important determinants of milk nutrient secretion is available in the review by Boyd and co-workers [34], where the authors concluded that level of milk nutrient secretion can be influenced by nutrient intake and endocrine stimulation. This hypothesis is supported by Tokach et al. [35], who demonstrated that energy intake by lactating sows greatly affects milk synthesis.

The impact of supplemental lipids on sow milk production and composition was investigated using 7 published studies [21, 23–26, 36, 37]. Because these studies used diverse estimation methods for milk production that included weigh-suckle-weigh and regression equations [38, 39]; sow milk production and nutrient output were re-estimated in all studies using prediction equations derived by Hansen et al. [40]. This re-analysis indicated that milk production averaged 8.4 kg/d (ranging from 6.7 to 9.8 kg/d) and milk fat output averaged 591 g/d (ranging from 401 to 814 g/d). There was a positive (250 g/d improvement) and consistent response (15 positive and 3 negative responses) on milk production when lipids were supplemented to diets. However, none of the studies reported significant responses. A greater (weighted average of 83.2 g/d) and more consistent response (all studies reported a positive responses and 4 were significant) was observed for milk fat output when lipids were supplemented to lactation diets.

**Fig. 1:**
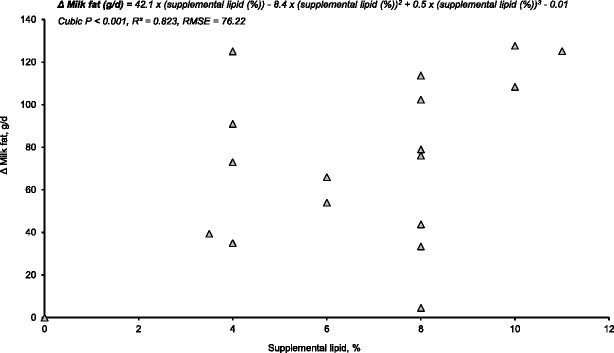
Effect of increasing lipid supplementation to lactation diets on the increase in milk fat output when compared with no added lipid diets. Symbols represent the improvements of supplemental lipids relative to no added lipid diets from results reported in 7 studies published from 1989 to 2015 [21, 23–26, 36, 37]. Observations with response means greater than 2 standard deviations from the mean were considered outliers and excluded from the analysis. Linear and non-linear (quadratic, cubic) models were compared using goodness-of-fit tests. Minimum Bayesian information criterion (BIC), minimum root mean square error (RMSE), and maximum coefficient of determination (R^2^) techniques were used to select the best-fit model. Weighted models were constructed with sample size (*n* = 4 to 33 sows per data point) as weight

Milk fat output may also be influenced by the age of the sow, ambient temperatures, level of lipid supplementation, and others. Averette et al. [22] observed that supplemental lipids improved milk fat content on d 2 and 3 of lactation in mature sows (parity 3 to 5), but not in parity 1 sows. Schoenherr et al. [23] concluded that the effect of supplemental lipids on milk fat output was greater during high ambient temperatures (32 °C; increased 90 g/d) than thermoneutral conditions (20 °C; 60 g/d). Figure 1 shows the increase in the amount of milk fat secreted as the level of supplemental lipid increased in lactation diets of different studies [21, 23–26, 36, 37]. Results from these studies were used to construct linear and non-linear (quadratic, cubic) models. Prediction equations for this and other variables were selected using goodness-of-fit tests that included minimum Bayesian information criterion (BIC), minimum root mean square error (RSME), and maximum coefficient of determination (R^2^). Supplemental lipids consistently improved milk fat output; however, the relationship between supplemental lipid level and the increase in milk fat output was not clear (cubic *P* < 0.001; R^2^ = 0.823; RSME = 76.22).

In summary, supplementation of lipids to lactation diets improved ADEI which seemed to be preferentially partitioned for milk, as indicated by the greater milk fat output and improved litter growth rate. The greater milk fat output positively influenced growth of the nursing litter. The impact of supplemental lipids on sow BW change and subsequent reproduction of sows is not clear from these studies.

### Lipid nutrition and subsequent reproduction of sows

In a commercial setting, the main objectives of a farrow-to-weaning operation are: 1) to maximize the number of healthy pigs weaned, 2) approximate their biological growth potential, and 3) to maximize the number of pigs weaned per sow life-time. Nutrition programs can be designed to prevent excessive body tissue mobilization of sows during lactation, thereby promoting life-time productivity of sows. This was demonstrated by Touchette et al. [41], who provided evidence that amino acid nutrition during lactation impacts the subsequent reproduction of sows. The authors increased lysine intake for parity 1 sows (from 32 to 52 g/d), which increased the number of pigs born in the subsequent cycle by 1.2.

The effect of supplemental lipids on the subsequent reproductive performance of sows was investigated in 6 studies (Table 3). Cox et al. [42] reported that supplemental lipids during lactation did not impact the WEI of sows housed under thermoneutral conditions, but reduced the WEI by 8.3 d (relative to no added lipid diets) for sows housed under high ambient temperatures (summer months). Later studies conducted under thermoneutral conditions reported modest positive responses for WEI and farrowing rate (percentage of sows that farrowed in the subsequent cycle relative to the number of weaned sows) [24, 30, 43].

**Table 3:** Effect of lipid supplementation to lactation diets on the change in subsequent wean-to-estrus interval (WEI) and subsequent farrowing rate^a,b^

					Normal temperatures		High temperatures	
Study	Reference^c^	Sows, n^d^	Source^e^	Level	Δ WEI, d	Δ Farrowing rate, %	Δ WEI, d	Δ Farrowing rate, %
1	[42]	64	Animal fat	10	−3.2		8.3*	
2	[43]	52	Dried-fat	10	2.4	0.7		
3	[24]	112	Corn oil	10	2	2.4		
4	[30]	36	Soy oil	5	0.2*			
5	[31]	84	A-V blend	2			1.4	10.0*
				4			1.3	14.1*
				6			1.2	7.2*
6	[32]	55	A-V blend	2				13.2*
				4				4.2
				6				13.2*
			CWG	2				11.6*
				4				5.8
				6				13.9*

^a^Summary of the results from 6 published studies from 1983 to 2012 that were not included in previous reviews and reported on supplemental dietary lipids in lactating sows diets

^b^Improvement of lipid supplementation relative to lactation diets containing no added lipids

^c^References are presented in chronological order

^d^n refers to the number of observations per treatment

^e^Animal-vegetable blend (A-V blend), choice white grease (CWG)

*P < 0.05

Improvements in subsequent farrowing rate of sows that were fed diets with added lipids were reported by Rosero et al. [31, 32]. These studies consistently reported improved farrowing rate (improvement by 10.3 %) when conducted during the summer heat stress. The authors observed that sows fed diets without added lipid had comparatively poor subsequent reproduction (farrowing rate < 75 %). Farrowing rate and culling rate (percentage of sows removed from the herd as cull sows relative to the number of sows weaned) each improved with the inclusion of at least 2 % supplemental lipid to lactation diets, either as choice white grease or animal-vegetable blend. In addition, the authors reported a linear improvement (from 13 to 14 total pigs born) in the subsequent litter of sows fed increasing doses of lipid (0, 2, 4 and 6 % added lipids) during lactation [32].

The studies conducted by Rosero and co-workers [31, 32] demonstrated that lipid supplementation during lactation resulted in a modest positive effect on sow lactation performance, but remarkably improved subsequent reproduction. These observations were the turning point in our understanding of sow lipid nutrition and led us to postulate that specific and essential fatty acids caused the improvement in reproduction, which has been proven to be true for the dairy cow [44, 45]. We hypothesized that the greatest benefit of added lipids during lactation was to improve subsequent reproduction by provision of essential fatty acids (EFA, linoleic acid, C18:2n-6; and α-linolenic acid, C18:3n-3) to correct a deficiency during lactation.

### Essential fatty acid nutrition during lactation

The essentiality of linoleic and α-linolenic acid (parental EFA) in animals is due to the absence of desaturase enzymes that are able to introduce double bounds distal from carbon 10 of octadecenoic acids. The lactating female secretes significant amounts of EFA in milk during lactation; fatty acids that are known to be essential for growth and development of the nursing litter [46, 47]. We recently suggested that the modern lactating sow secretes EFA in the milk, even if this results in mobilization from body adipose reserves [37]. It was postulated that excessive secretion of dietary and mobilized EFA in milk could, at some point, result in EFA deficiency to the extent that reproduction could be impaired. Further, this deficiency could be corrected by specifically supplementing EFA. The involvement of EFA in reproduction processes suggests that potential EFA deficiencies could be related with infertility of females, which was proven to be true [48], as discussed below.

#### Metabolism of essential fatty acids

**Fig. 2:**
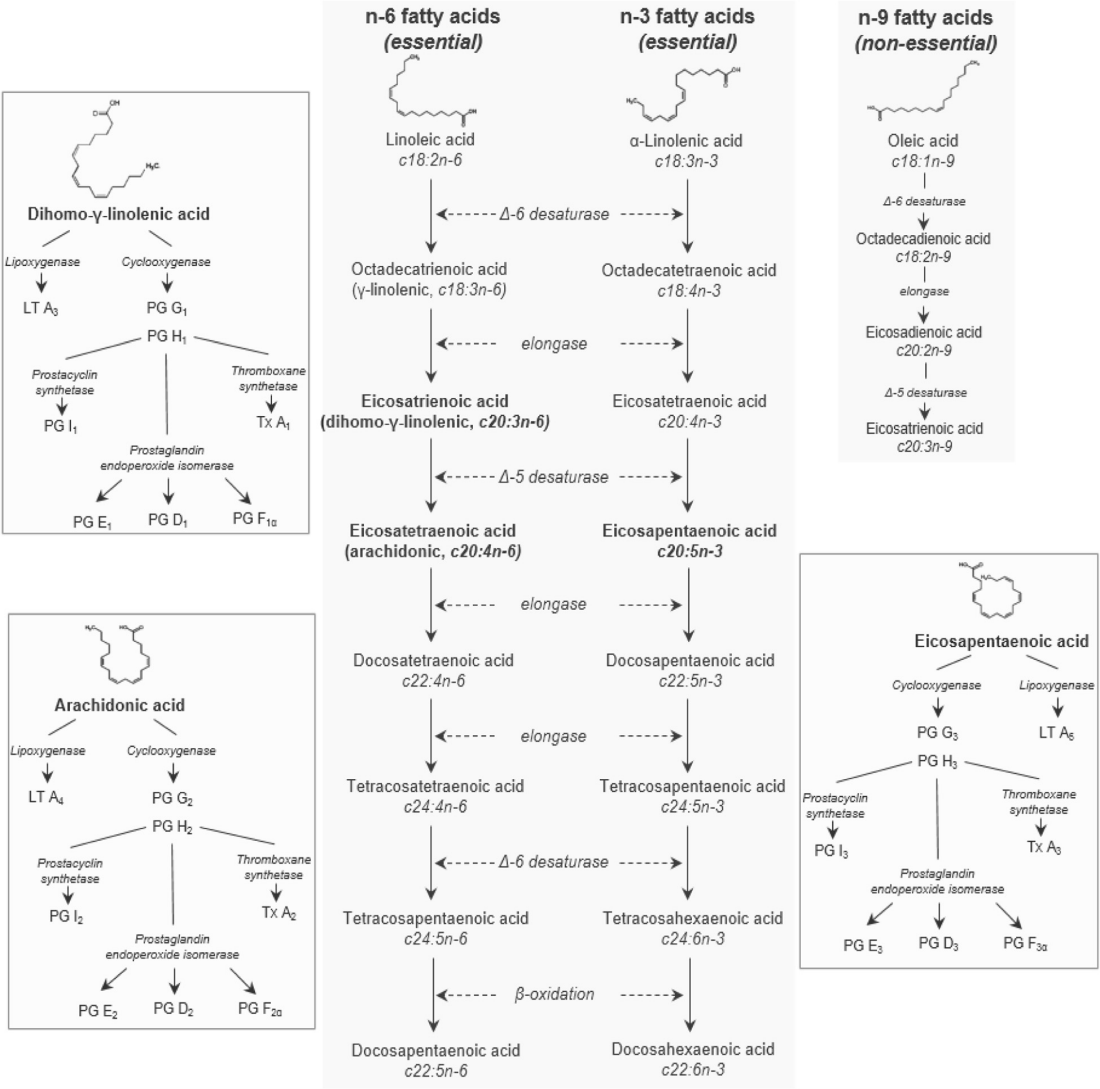
Schematic overview of the elongation of the parent essential fatty acids (linoleic and α-linolenic acid) to long chain polyunsaturated fatty acids and conversion to eicosanoids. Dietary octadecenoic acids (parent fatty acids) are converted to long chain PUFA by microsomal desaturase and elongase enzymes that are shared by the n-3 and n-6 fatty acids. [49–51]. The n-3 and n-6 fatty acids (dihomo-γ-linolenic, arachidonic, and eicosapentaenoic acid) are precursors of diverse eicosanoids by different pathways, in which enzymes such as cyclooxygenase, lipoxygenase, endoperoxide isomerase, and others are involved [68]. (Adapted with permission from: [69])

The two essential families of fatty acids are the “omega-3” or n-3, and the “omega-6” or n-6. Animals can convert dietary octadecenoic acids (parent fatty acids: linoleic and α-linolenic acid) to long chain PUFA (LC-PUFA) by microsomal desaturase and elongase enzymes (Fig. 2) [49, 50]. In the n-6 family, linoleic acid can be converted into γ-linolenic (18:3n-6), dihomo-γ-linolenic (20:3n-6), arachidonic (20:4n-6) and other fatty acids. In the n-3 family, α-linolenic acid (18:3n-3) can be converted to eicosatetraenoic (20:4n-3), eicosapentaenoic (20:5n-3), docosahexaenoic acid (22:6n-3) and other important LC-PUFA [51]. Conversions of octadecenoic acids to LC-PUFA are mediated by enzymes that are shared by the n-3 and n-6 fatty acids. These enzymes have greater affinity for n-3 fatty acids than for n-6 fatty acids. Thus, conversion of n-6 fatty acids to LC-PUFA is reduced by increasing the availability of n-3 fatty acids (lowering the n-6: n-3 fatty acid ratio). The n-3 and n-6 fatty acids (dihomo-γ-linolenic, arachidonic, and eicosapentaenoic acid) are precursors of diverse eicosanoids by different pathways, in which enzymes such as cyclooxygenase, lipoxygenase, endoperoxide isomerase, and others are involved. Eicosanoids include prostaglandins (of series 1, 2, and 3), leukotrienes and thromboxanes [51].

#### The balance of EFA during lactation

**Fig. 3:**
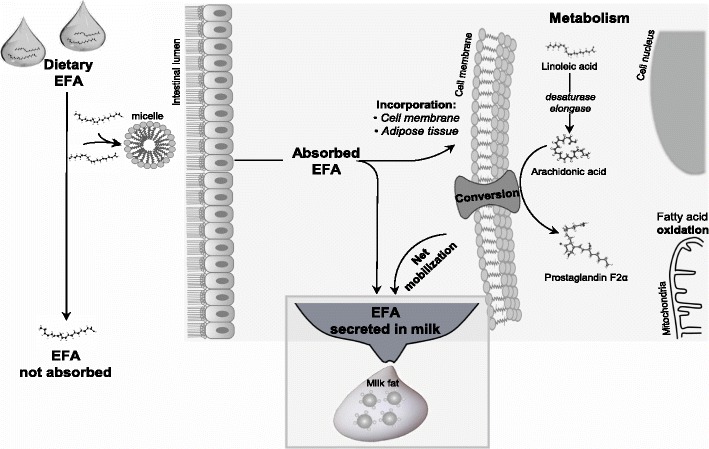
Simplified overview of the source, partitioning, and net balance (intake minus milk output) of essential fatty acids (EFA, linoleic and α-linolenic acid) fed to lactating sows. Fatty acids absorbed into the body can be deposited into body tissues (e.g. adipose tissue, cell membranes), elongated to long-chain PUFA, converted to active metabolites [68], or oxidized for energy. The greatest proportion of absorbed EFA is expected to be extracted by the mammary gland and secreted in milk [33]

A simplified overview of the source, partitioning, and net balance of EFA during lactation is illustrated in Fig. 3. We expect that the likelihood for a negative balance is greatest during lactation because EFA secretion in milk would far surpass the daily intake, thereby requiring tissue mobilization. The balance of EFA during lactation represents the inflow (intake minus fatty acids not absorbed) minus the outflow of EFA. Absorbed EFA may be deposited into body tissues (e.g. adipose tissue, cell membranes, etc.), elongated to LC-PUFA, converted to active metabolites (e.g. eicosanoids), or oxidized for energy. The greatest proportion of absorbed EFA is expected to be extracted by the mammary gland and secreted into milk [33]. Estimation of the balance of EFA is important to determine if deficiency of EFA during lactation is likely; the latter being a pre-requisite for a dose-response assay. A negative EFA balance during lactation indicates a net mobilization of EFA from body tissues and the progressive decline in body EFA pool size is expected to eventually disturb fertility of sows.

**Fig. 4:**
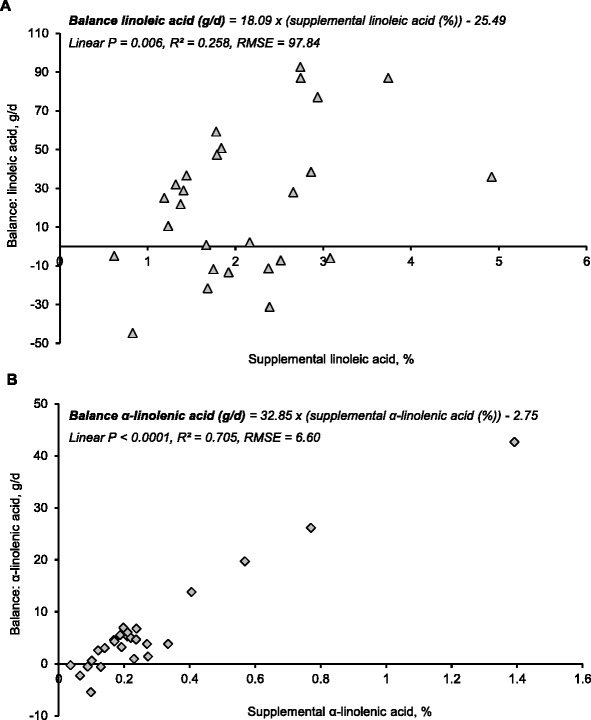
Effects of increasing linoleic (**a**) and α-linolenic acid (**b**) supplementation to lactation diets on the balance (net uptake minus output in milk) of essential fatty acids during lactation. Symbols represent the estimated EFA balance using results reported in 6 studies published from 1977 to 2015 [26, 37, 52–55]. Observations with response means greater than 2 standard deviations from the mean were considered outliers and excluded from the analysis. Linear and non-linear (quadratic, cubic) models were compared using goodness-of-fit tests. Minimum Bayesian information criterion (BIC), minimum root mean square error (RMSE), and maximum coefficient of determination (R^2^) techniques were used to select the best-fit model. Weighted models were constructed with sample size (*n* = 3 to 33 sows per data point) as weight

Rosero et al. [37] observed that for sows fed diets without supplemental lipids, the amount of EFA secreted in milk (90 g/d of linoleic and 4 g/d of α-linolenic acid) was greater than the estimated intake of EFA throughout lactation (78 g/d of linoleic and 4 g/d of α-linolenic acid). A negative balance of linoleic acid (as low as −12 g/d) was estimated for these sows. This estimate of apparent negative balance was expected to be conservative because we could not account for EFA conversions and endogenous EFA loss was not estimated. The EFA balance during lactation was further investigated using 6 published studies that provided sufficient data regarding fatty acid composition (both diet and milk), feed intake and litter growth performance [26, 37, 52–55]. From this multi-trial analysis, we estimated that apparent balance of linoleic acid during lactation was −25.49 g/d for sows fed diets without supplemental linoleic acid. Similarly, a negative balance of α-linolenic acid of −2.75 g/d was estimated when sows were fed diets without supplemental α-linolenic acid. Increasing supplemental EFA greatly increased the balance of linoleic (linear *P* = 0.006; R^2^ = 0.258; RMSE = 97.84; Fig. 4a) and α-linolenic acid (linear *P* < 0.0001; R^2^ = 0.705; RMSE = 6.60 Fig. 4b) during lactation.

Despite the essentiality of EFA during lactation, current dietary recommendations for sows specify a low requirement for linoleic acid (0.1 % of the diet or 6 g/d, assuming a feed intake of 6.28 kg/d) and no requirement minimum or maximum estimate for α-linolenic acid is specified [56]. Compared with the significant amounts of linoleic acid secreted in milk of sows fed diets without supplemental EFA (90 g/d) [37], the current recommendation estimate of 6 g/d appears too low. Based on the minimum amount of linoleic acid secreted in milk, it is suggested that the provision of at least 100 g/d of linoleic acid will ensure adequate consumption to prevent a potential negative balance during lactation.

#### The role of EFA in the sow and litter performance during lactation

A desired outcome of feeding sows diets supplemented with n-6 and n-3 fatty acids (using lipids from either plant or marine origin) is also to increase the concentrations of LC-PUFA in neonatal piglet tissues. The potential benefits of these fatty acids include enhanced neural development, improved immune response, and enhanced protective function of the intestine [46, 57, 58]. Indeed, Farmer et al. [36] and Yao et al. [59] demonstrated that supplementation of n-3 fatty acids (flaxseed meal or oil) to lactating sow diets increased the immune response of nursing piglets and improved piglet survival.

Notwithstanding the apparent deficit in linoleic acid intake for lactation, addition of n-3 fatty acids to sow diets has been of greater interest because common diets contain limited levels of these fatty acids. There is strong evidence to suggest that n-3 LC-PUFA play important roles in the cognitive and neural development and may benefit health of piglets [47]. Although conversion from α-linolenic acid to LC-PUFA seems to be limited in mammals [50], some researchers reported that supplemental flaxseed oil (rich in α-linolenic acid) to lactating sow diets resulted in increased concentrations of n-3 LC-PUFA in piglet’s brain [60]. The potential benefits of supplementing α-linolenic acid to sows on litter performance are still controversial as results of published studies are inconsistent [61].

Increasing relative dietary concentrations of α-linolenic acid (lowering the n-6: n-3 fatty acid ratio) results in decreased conversion of linoleic acid to LC-PUFA and increased conversion of α-linolenic acid to its derivatives. These EFA are competitive substrates for the desaturase enzyme (Δ6) that has greater affinity for α-linolenic acid [49]. Recently, Yao et al. [59] concluded that altering the n-6:n-3 fatty acid ratio in lactating sow diets influenced the concentrations of immunoglobulins in sow colostrum and piglet plasma. The authors speculated that increasing availability of n-3 fatty acids can decrease the production of arachidonic acid-derived eicosanoids such as prostaglandin E_2_, which can negatively impact the production of immunoglobulins. This research suggests that the lactation n-6:n-3 fatty acid ratio is highly important and warrants further work, especially to assess the immune response of lactating sows and piglets.

#### The role of EFA in the subsequent reproduction of sows

Although supplemental EFA was demonstrated to benefit subsequent reproduction of the dairy cow (discussed later) [44, 45], little evidence exists for the modern lactating sow [56]. Recently, Smits et al. [62] suggested that supplementation of n-3 fatty acids during lactation, using fish oil as source, increased subsequent litter size. To our knowledge, the only study that investigated the impact of supplemental linoleic acid on lactating sow performance and subsequent reproduction was conducted almost 4 decades ago by Kruse et al. [52]. In this study, a total of 9 sows (3 sows per treatment) were fed increasing amounts of linoleic acid (30, 75 and 125 g/d by supplementing 0, 2 and 4 % soybean oil, respectively) and the response of sows was collected over 3 consecutive parities. The authors reported no benefit from supplemental linoleic acid on lactating sow performance or subsequent reproduction of sows. The authors suggested that supplemental linoleic acid beyond that provided by a practical diet without added lipids seemed to be sufficient for the low-productivity sow (weaned pigs *=* 7.1 and litter growth rate = 1.36 kg/d). Ignoring the fact that the number of sows involved were woefully inadequate for such a test, we suggest that these findings do not hold true for the greater productivity level of the modern lactating sow because litter-size (pigs born and weaned) presently is almost twice that studied.

We conducted a dose-response study to determine the levels of both parent EFA required by the modern lactating sow for maximum subsequent reproduction [48]. In this study, a total of 480 lactating sows (equally balanced by parity 1, and 3 to 5, P3+) were assigned randomly to a 3 × 3 factorial arrangement plus a control diet without added lipid. Factors involved linoleic (2.1, 2.7 and 3.3 %) and α-linolenic acid (0.15, 0.30 and 0.45 %), which were obtained by adding 4 % of mixtures of canola, corn, and flaxseed oils to diets. This study was designed to investigate the dose response to linoleic and α-linolenic acid because these fatty acids are precursors of compounds with opposing functions and increasing the availability of one of these fatty acids decreases the metabolism and physiological functions of the other [49]. Therefore, we hypothesized that supplementation of each fatty acid potentially benefits sow reproductive efficiency through different mechanisms. In this study, the response of sows was assessed using multiple criteria that included the percentage of sows that returned to estrus, maintenance of pregnancy, and litter size in the subsequent reproductive cycle. A minimum requirement for each essential fatty acid was anticipated to maximize the response for one or more of the various criteria.

Although supplemental linoleic acid improved subsequent reproduction of parity 1 sows, the beneficial effects of EFA were more evident for aging sows (P3+). This may be due to a progressive reduction in the body EFA pool over successive lactations. In other words, lactation expenditure was not adequately replenished during pregnancy. Noticeably, P3+ sows fed lactation diets containing low levels of EFA (<2.7 % linoleic acid, < 0.45 % α-linolenic acid) had a poor subsequent farrowing rate (76 %) and elevated culling rate (25 %; proportion of sows removed from the herd as culls) (Table 4). It is likely that these sows were under a profound negative EFA balance during lactation. Under these conditions, high levels of supplemental linoleic acid (≥2.7 %) or α-linolenic (>0.30 %) improved farrowing (>83.6 %) and reduced culling rate (<16.7 %).

**Table 4:** Effects of increasing essential fatty acid supplementation to lactation diets on the subsequent reproductive cycle of mature sows (parity 3–5 sows)^a,b^

Linoleic acid, %		2.1			2.7			3.3			
α-Linolenic acid, %		0.15	0.30	0.45	0.15	0.30	0.45	0.15	0.30	0.45	
Item^1^	Control^c^										*SEM*
Sows weaned, n	24	24	24	23	23	24	24	22	24	23	
Sows bred: weaned, %	91.7	87.6	96.0	95.7	87.0	87.5	100.0	86.4	95.8	87.5	5.7
Wean-to-estrus interval,^2^ d	4.6^e^	5.0^e^	4.1^de^	3.7^d^	4.4^de^	4.3^de^	4.6^e^	4.2^de^	4.0^de^	3.8^d^	0.1
Farrowing rate,^3^ %	79.2^de^	74.9^e^	75.8^e^	95.7^d^	87.3^de^	83.7^de^	95.9^d^	86.8^de^	83.6^de^	87.4^de^	7.2
Culling rate,^4^ %	16.7^de^	25.0^e^	25.0^e^	4.3^d^	13.0^de^	16.7^de^	4.2^d^	13.6^de^	4.3^d^	13.0^de^	6.0

^a^Modified from [48]

^b^Diets supplemented to lactation sows were isocaloric and contained 4 % added lipids obtained by blending canola, corn and flaxseed oils

^1^Supplemental linoleic × α-linolenic acid interactions were not detected for any of the variables (*P* > 0.10)

^c^Control diet was calculated to contain 1.3 % linoleic and 0.07 % α-linoleic acid from diet ingredients

^2^Linear tendency for supplemental α-linolenic acid (linear *P* = 0.098, lack of fit *P* = 0.699)

^3^Proportion of sows farrowed: weaned; linear tendency for supplemental α-linolenic acid (linear *P* = 0.080, lack of fit *P* = 0.100)

^4^Proportion of cull sows: weaned; linear tendency for supplemental α-linolenic acid (linear *P* = 0.079, lack of fit *P* = 0.662)

^d,e^Within a row, estimated means without a common superscript differ (*P* < 0.05)

We observed responses to the main effects of α-linolenic and linoleic acid dose. A minimum provision of 0.45 % of α-linolenic acid was the most effective dietary treatment in causing rapid return to estrus (sows bred: sows weaned = 94.2 %; wean-to-estrus interval = 4.0 d) and achieved the highest retention of pregnancy (sows pregnant: sows bred = 98 %), but it did not appear to influence subsequent litter size. Moreover, supplemental linoleic acid elicited a linear effect on the number of total pigs born (linear *P* = 0.075; lack-of-fit *P* = 0.496; 13.2, 13.8, and 14.0 total pigs born for 2.1, 2.7 and 3.3 % linoleic acid, respectively) in the subsequent cycle of sows [48].

The different responses for both parent EFA confirmed our hypothesis that a minimum provision of each EFA was required to maximize reproductive efficiency for the various criteria. On this basis, we concluded that a minimum dietary intake of 10 g/d of α-linolenic acid, simultaneous with a minimum of 100 g/d of linoleic acid should be provided to > 90 % of the sows (considering large variability in feed intake of sows); thereby, collectively maximizing the subsequent reproduction of sows through a multiple of mechanisms (rapid return to estrus, high maintenance of pregnancy and increased litter size).

We recognize the importance of the optimum n-6: n-3 fatty acid ratio during lactation, but suggest that this is only meaningful when the absolute amount of both parent EFA are not significantly deficient. This was evident in the described study [48], in which diets with a similar n-6: n-3 fatty acid ratio of 7 resulted in different subsequent reproductive outcomes (e.g. 75 vs. 87 % farrowing rates). Further investigation of the optimum n-6: n-3 fatty acid ratio during lactation is warranted; but this has to be established after satisfying EFA needs.

#### Validation of the linoleic acid requirement

Data from 3 sow studies that were conducted in the same research farm under heat stress and using similar methodology (genetic line, feeding system, etc.), allowed us to investigate further the impact of supplemental linoleic acid on the subsequent reproduction of sows [31, 32, 48]. This multi-trial analysis focused on the impact of supplemental linoleic acid because the sources of lipid used in these studies had relatively high levels of linoleic acid (animal-vegetable blend 27 %; choice white grease = 13 %) providing a wide range of linoleic acid intake, but this was not the case for α-linolenic acid (animal-vegetable blend 1.1 %; choice white grease = 0.5 %). This analysis investigated the subsequent reproduction of a total of 543 mature sows (parities 3 to 5). The 3 studies included groups of sows fed diets without added lipids (linoleic acid intake averaged 84.4 ± 20.3 g/d) and these are presented in the analysis as no added lipid treatment (*n* = 84). For the multi-trial analysis, sows fed lipid supplemented diets were equally balanced into groups according to their total linoleic acid intake during lactation. Number of linoleic acid intake groups and sample size within each group were chosen to maximize the statistical power for the analysis of reproduction responses.

**Fig. 5:**
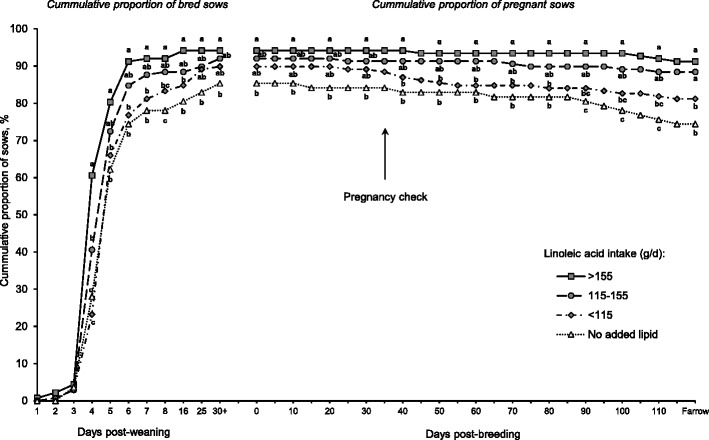
Effects of linoleic acid intake during lactation on the subsequent reproductive cycle of sows. Symbols (*n* = 84 sows fed diets containing no added lipids and *n* = 152, 163, and 144 sows for < 115, 115 to 155, and > 155 g/d of linoleic acid intake, respectively) represent the cumulative proportion of bred and pregnant sows (relative to the number of sows weaned) (SEM = 2.9). This analysis included a total of 543 mature sows (parities 3 to 5) from 3 studies [31, 32, 48]. Sows fed diets containing no added lipids consumed 84.4 ± 20.3 g/d of linoleic acid. Increased consumption of linoleic acid (> 115 g/d) during lactation improved the proportion of weaned sows that were bred (> 88 %; day 8 post-weaning *P* = 0.024) and farrowed in the subsequent cycle (> 88 %; *P* = 0.007). Data were analyzed by logistic regression using the GLIMMIX procedure of SAS using a logit link function. Means represented by symbols without a common letter are different (*P* < 0.05)

Figure 5 illustrates the impact of linoleic acid intake during lactation on subsequent reproduction of sows after weaning. For this illustration, sows were equally balanced in 3 groups according to their linoleic acid intake during lactation (*n* = 137 to 138 sows per linoleic acid intake group). The proportion of weaned sows that were bred (85.4 %) and farrowed in the subsequent cycle (74.4 %) was reduced when they consumed diets without supplemental lipids during lactation. The subsequent reproduction of sows was improved with linoleic acid supplementation during lactation. A high proportion of weaned sows were bred (>88 %; day 8 post-weaning *P* = 0.024) and farrowed (>88 %; *P* = 0.007) when they consumed more than 115 g/d of linoleic acid during lactation. Remarkably, it was also noted that the elevated farrowing rate was related to the improved ability of sows to maintain pregnancy (>96 % of sows maintained pregnancy if they consumed more than 115 g/d of linoleic acid). The ability of sows to maintain pregnancy was reduced (<90 % of bred sows) when they consumed less than 115 g/d of linoleic acid during lactation but especially when they consumed diets without added lipids.

**Fig. 6:**
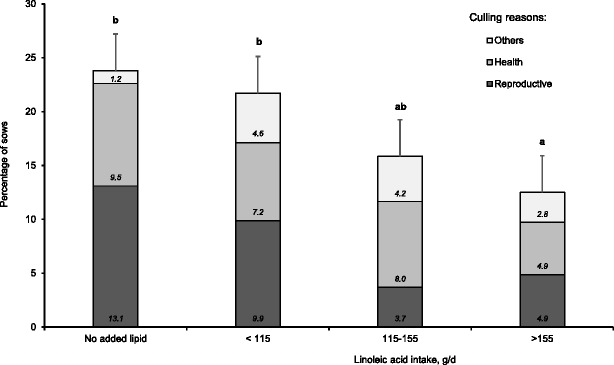
Effects of linoleic acid intake during lactation on culling rate. Bars represent the percentage of sows removed from the herd (relative to the number of weaned sows) as cull sows (*n* = 84 sows fed diets containing no added lipids and *n* = 152, 163, and 144 sows for < 115, 115 to 155, and > 155 g/d of linoleic acid intake, respectively). This analysis was performed using mature sows (parities 3 to 5) from 3 studies [31, 32, 48]. Sows fed diets containing no added lipids consumed 84.4 ± 20.3 g/d of linoleic acid. Increased consumption of linoleic acid during lactation increasingly reduced the number of sows removed from the herd as culls (*P* = 0.085). Data were analyzed by logistic regression using the GLIMMIX procedure of SAS using a logit link function. Means represented by bars without a common letter are different (*P* < 0.05)

The reduced number of sows returning to estrus after weaning and reduced ability of sows to maintain pregnancy after insemination, when they consumed less than 115 g/d of linoleic acid or diets without supplemental lipids, resulted in high culling rates (Fig. 6). Increased consumption of linoleic acid during lactation progressively reduced the number of sows removed from the herd as culls (*P* = 0.085). The improvement in culling rate was related to a reduced number of sows removed from the herd due to reproductive failure that included sows not returning to estrus, sows returning to estrus after breeding, and pregnancy loss.

**Fig. 7:**
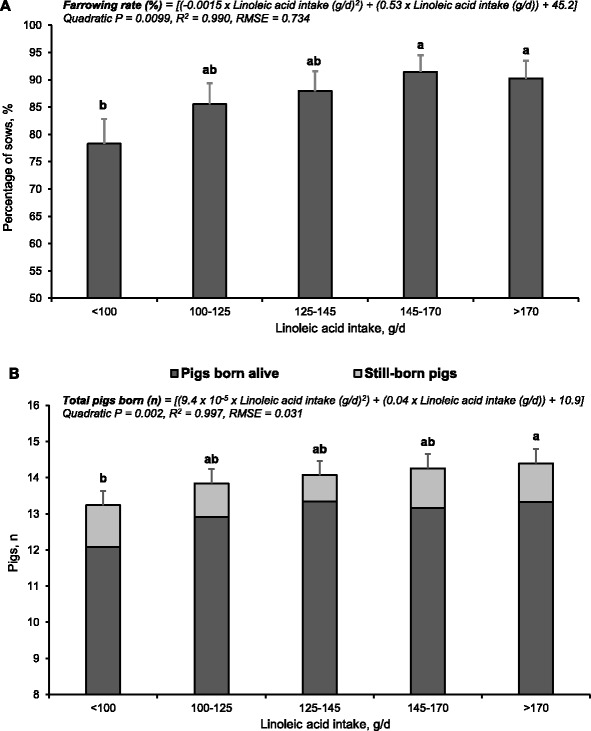
Effects of linoleic acid intake during lactation on (**a**) the farrowing rate and (**b**) total pigs born in the subsequent cycle of sows. In Fig. 7 (**a**), bars represent the percentage of sows that farrowed in the subsequent cycle relative to the number of weaned sows ± SEM (*n* = 82 to 83 sows per linoleic acid intake (g/d) group). In Fig. 7 (**b**), bars represent the number of pigs born alive and still-born pigs ± SEM (*n* = 70 sows per linoleic acid intake group). This analysis was performed using mature sows (parities 3 to 5) from 3 studies [31, 32, 48]. Linear and non-linear (quadratic, cubic) models were compared using goodness-of-fit tests. Minimum Bayesian information criterion (BIC), minimum root mean square error (RMSE), and maximum coefficient of determination (R^2^) techniques were used to select the best-fit model. Means represented by bars without a common letter are different (*P* < 0.05)

We used this multi-trial data set to estimate the minimum requirement for linoleic acid during lactation, to maximize subsequent pig output in mature sows. The method involved a dose-response assay, using the key reproductive parameters subsequent farrowing rate (Fig. 7A) and total pigs born (total fully-formed pigs, Fig. 7B). The dose-response relationship between the parameters and linoleic acid intake was established using 5 sow groups divided equally for total linoleic acid intake during lactation (farrowing rate, *n* = 82–83 sows; total pigs born, *n* = 70 sows per linoleic acid intake group).

For subsequent farrowing rate and total pigs born, increasing linoleic acid intake elicited a similar dose response form (curvilinear function). Subsequent farrowing rate (%) was described by: y (%) = [(−1.5 × 10^−3^ × linoleic acid intake (g/d)^2^) + (0.53 × linoleic acid intake (g/d)) + (45.2); quadratic *P* = 0.002, R^2^ = 0.997, RMSE = 0.031]. Total pigs born was described by: Pigs (n) = [(9.4 × 10^−5^ × linoleic acid intake (g/d)^2^) + (0.04 × linoleic acid intake (g/d)) + (10.94); quadratic *P* = 0.002, R^2^ = 0.997, RMSE = 0.031]. The greatest marginal improvement for subsequent farrowing rate (7 %) and total pigs born (0.60 pigs) was observed for sows that consumed more than 100 g/d of linoleic acid (vs. <100 g/d). We previously estimated, without the benefit of this large data set, that a minimum of 100 g/d of linoleic acid/d is required for near maximum reproductive performance (>90 % of sow population) [48].

**Fig. 8:**
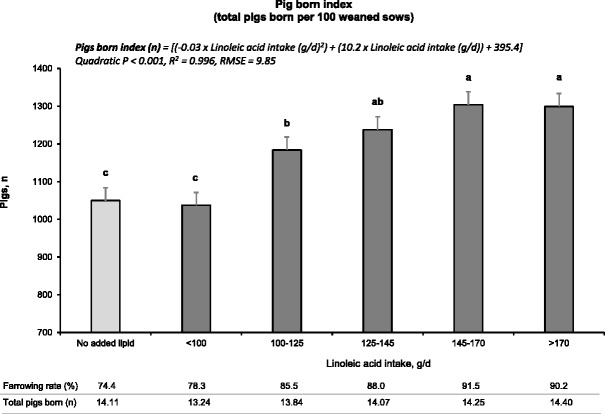
Impact of linoleic acid intake during lactation on the pigs born index. This variable represents the number of fully-formed pigs born per 100 weaned sows and was calculated by multiplying the subsequent farrowing rate (sows farrowed: weaned) and the number of pigs born in the subsequent cycle. Sows fed diets containing no added lipids consumed 84.4 ± 20.3 g/d of linoleic acid. Linear and non-linear (quadratic, cubic) models were compared using goodness-of-fit tests. Minimum Bayesian information criterion (BIC), minimum root mean square error (RMSE), and maximum coefficient of determination (R^2^) techniques were used to select the best-fit model. Means represented by bars without a common letter are different (*P* < 0.05)

Based on this enlarged data set, the linoleic acid dose that elicited the maximum improvement in farrowing rate was slightly greater than required to maximize total pigs born (Fig. 7). We calculated a ‘pigs born per 100 sows weaned index’ to better quantify the impact of total linoleic acid intake during lactation on the number of pigs produced in the subsequent cycle (Fig. 8). This index is a composite of farrowing rate and total pigs born and represents the total fully-formed pigs born per 100 weaned sows. The dose-response relationship of pigs born index on linoleic acid intake integrates the outcome for both parameters to deliver a more meaningful result that best represents the reproductive outcome. This composite expression facilitates economic evaluation. The index was described by: y (n) = [(−0.03 × linoleic acid intake (g/d)^2^) + (10.2 × linoleic acid intake (g/d)) + (395.4); quadratic *P* < 0.001, R^2^ = 0.996, RMSE = 9.85].

The clarifying nature of the pigs born index is illustrated by comparing the no added lipid treatment to the five added linoleic acid doses. The former group consumed an average of 84.4 ± 20.3 g linoleic acid/d. Although litter-size was relatively high (14.11 pigs/litter) and comparable to the 125–145 g/d linoleic acid intake sow group, farrowing rate was relatively low (74 %), in contrast to the latter group (88.0 %). This suggests that sows in the no added lipid group, that were able to maintain pregnancy, had a high number of pigs born. However, this is misleading because 14 % fewer sows maintained pregnancy. The pigs born index for sows fed diets without added lipids was 1050 pigs, which was comparable to the index of sows consuming <100 g/d of linoleic acid (1037 pigs), but not those in the 125–145 g/d group (1238 pigs). This integration of pregnancy maintenance with litter-size delivered is the preferred descriptor of EFA response.

Based on the dose-response curve that is presented in Fig. 8, we estimate that the near maximum response to total linoleic acid intake is achieved if sows consume a minimum of 125 g linoleic acid/d during lactation. The greatest marginal difference in pigs born index, for the 5 dose response curve, resulted when moving from <100 g/d to 100–125 g/d (147 pigs); the marginal difference when moving from 100–125 to 125–145 g/d being 54 pigs/100 sows weaned. The dose-response assay (Fig. 8) is the first estimate, to our knowledge, of the linoleic acid requirement for reproduction in any species. This estimate will vary with (a) age of sow; being greater for aging sows as compared to younger sows, and (b) life-cycle replenishment during pregnancy; diets composed of corn and corn distillers grains being advantageous to those composed of milo and wheat middlings.

For practical application, we propose that the minimum requirement of linoleic acid intake should be based on the pigs born index (fully-formed pigs). The equation in Fig. 8 can be used to derive a financial optimum intake of linoleic acid during lactation. Proper implementation of the 125 g/d estimate requires knowledge of seasonal lactation intake and the variance around intake so that the minimum linoleic acid intake (g/d) is achieved for perhaps 90 to 95 % of the sows in the population. For example, if the bottom 10 % of the sows are predicted to consume 4.2 kg/d (ADFI = 5.5 ± 1.0 kg/d) under heat stress conditions, and if a minimum of 125 g/d linoleic acid is desired, then the dietary specification would be 2.96 % linoleic acid. We anticipate that the lower lactation intake for mature sows, for which the estimate is intended, is at or above that for the lowest 10 % of the sows in this example.

#### Lactation EFA and possible mechanisms in cattle

The present review presents an intriguing and novel finding that supplemental EFA during lactation benefits the subsequent reproduction of the modern sow. Dose-response studies allowed us to estimate the minimum requirement of linoleic and α-linolenic acid. Although it is more difficult to establish minimum requirements in cattle because of the complication of the rumen and microbial metabolism of fatty acids, extensive research demonstrated that lactation EFA is an effective nutritional strategy to improve the fertility of females. In an extensive review, Staples et al. [63] concluded that supplemental lipids improved reproduction function and fertility in cattle, and suggested that positive responses were the result of providing supplemental EFA. The possible mechanisms that have been proposed included: nutraceutical regulation post-partum, modulation of follicle development, improved embryonic quality, increased concentrations of hormones important in reproduction (e.g. prostaglandins, progesterone), and pregnancy recognition and maintenance via cell signaling [64].

**Fig. 9:**
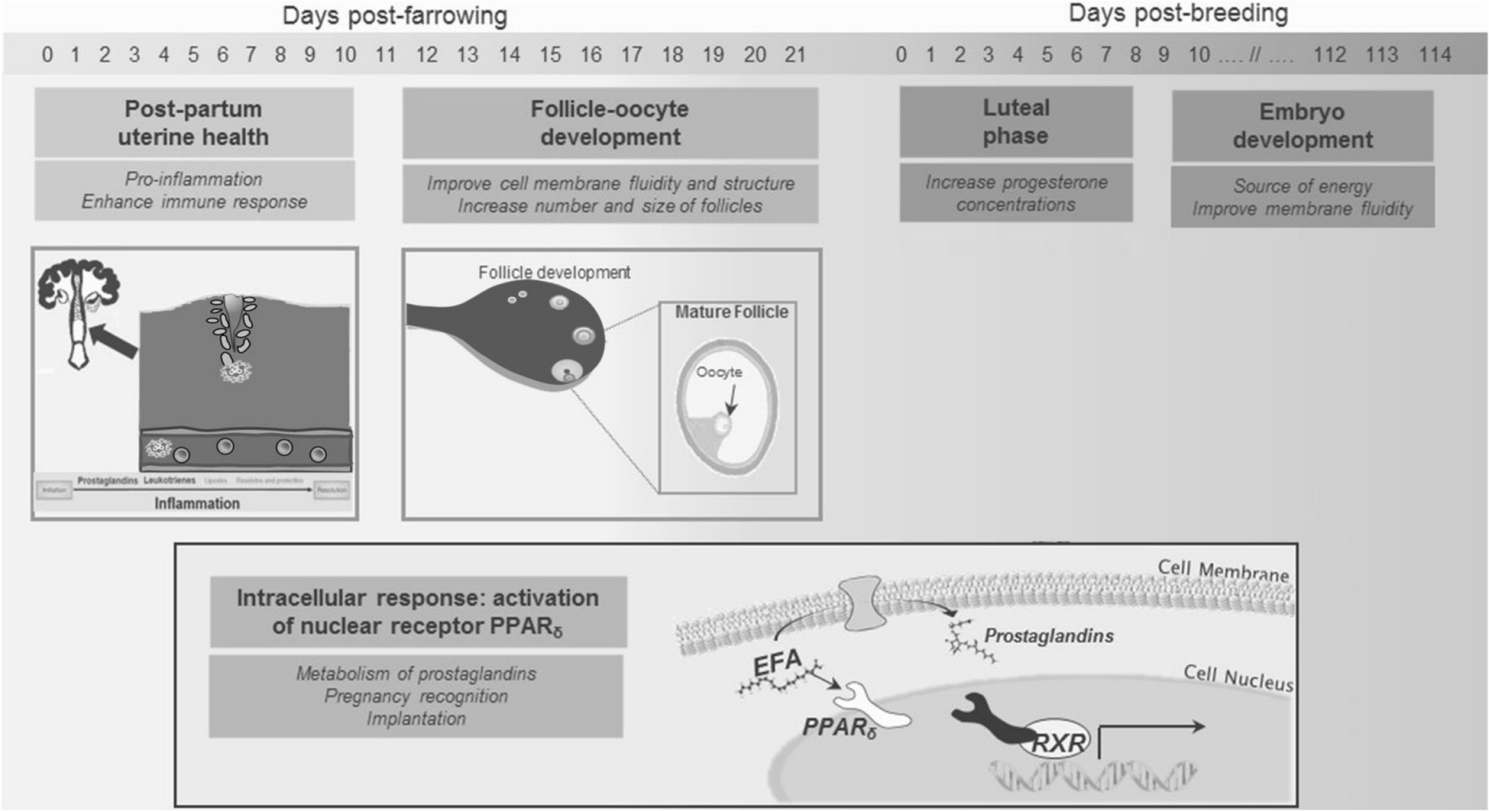
Schematic illustration of the critical roles of essential fatty acids on the reproduction of sows. Lactation EFA enhances uterine secretion of prostaglandin F2α, which could reduce severity and incidence of uterine disease post-partum [44, 65]. Lactation EFA enhances follicle development and growth and improves oocyte quality in cattle [44, 67]. EFA activates the peroxisome proliferator-activated receptor δ (PPAR-δ), which influences the metabolism of prostaglandins and is involved in pregnancy recognition and implantation processes [44]

Figure 9 illustrates possible mechanisms of supplemental EFA during lactation that positively impact the subsequent reproduction. For the purpose of the present review, we briefly discuss potential mechanisms of EFA when supplemented to lactation diets. Feeding a protected lipid (rich in linoleic acid) during early lactation of cattle reduced the severity and incidence of uterine disease postpartum (e.g. retained placenta, metritis) and this was related with enhanced uterine secretion of prostaglandin F2α [44, 65]. Prostaglandin F2α is synthesized by the endometrium using linoleic acid as a precursor. In 4 experiments (using 435 to 910 cows in each experiment), Lopes et al. [66] demonstrated that supplementation of rumen-protected lipid (40 % linoleic and 3 % α-linolenic acid) to diets of lactating cows improved pregnancy rates at d 28 post insemination by more than 12 % when compared with cows fed diets with no added lipid. Moreover, oocyte membrane fluidity is influenced by its phospholipid content and it improves with unsaturated fatty acids. Supplemental EFA during lactation has also been related to enhanced follicle development and growth and improved oocyte quality in cattle [44, 67]. Furthermore, Santos et al. [44] suggested that supplemental EFA activates the peroxisome proliferator-activated receptor δ (PPAR-δ), which influences the metabolism of prostaglandins and is involved in the pregnancy recognition and implantation processes.

We conclude that supplemental EFA during lactation corrects the negative EFA balance and positively impacts the ability of sows to achieve and maintain pregnancy and improves subsequent litter size. Supplemental EFA during lactation seems to be increasingly important with advancing sow age and is expected to be more important under conditions of heat stress. Feeding programs for the modern lactating sow should be designed to provide a minimum dietary intake of 10 g/d of α-linolenic acid, simultaneous with a minimum of 125 g/d of linoleic acid to > 95 % of the sows; thereby, collectively achieving a maximum sow reproductive efficiency through multiple mechanisms that include rapid return to estrus, high maintenance of pregnancy and improved subsequent litter size.

## Conclusions

This review shows that supplemental lipids improve caloric intake of lactating sows, which improves milk fat output and litter growth rate. Most importantly, supplemental lipids resulted in a remarkable improvement in return to estrus after weaning, maintenance of subsequent pregnancy and subsequent litter size. We contend that supplemental EFA during lactation corrects a negative EFA balance and this improved the fertility of the modern sow; a phenomenon that seems to be increasingly important with advancing sow age. Feeding programs for the modern lactating sow should be designed to provide a minimum dietary intake of 10 g/d of α-linolenic acid, simultaneous with a minimum of 125 g/d of linoleic acid provided to > 95 % of the sows; thereby, achieving a maximum sow reproductive efficiency through multiple mechanisms that include rapid return to estrus, high maintenance of pregnancy and large subsequent litter size in mature sows, that appear to be especially susceptible to EFA deficiency.

## Abbreviations

ADEI: average daily energy intake
A-V: animal-vegetable
BW: body weight
CWG: choice white grease
DE: digestible energy
EFA: essential fatty acids
FFA: free fatty acids
LC-PUFA: long-chain polyunsaturated fatty acids
MCT: medium chain triglycerides
ME: metabolizable energy
U:S: unsaturated to saturated fatty acids ratio
WEI: wean-to-estrus interval
